# Different Acupuncture Combined With Three‐Step Analgesic Therapy in the Treatment of Bone Metastatic Cancer Pain: A Network Meta‐Analysis Based on Randomized Controlled Trials

**DOI:** 10.1155/prm/7404872

**Published:** 2026-09-02

**Authors:** Bofeng Zhao, Weiming Cao, Peijie Qin, Shiheng Wang

**Affiliations:** ^1^ School of Traditional Chinese Medicine, Shandong Medical and Pharmaceutical University, Yantai, China; ^2^ Acupuncture Department 1, Yueyang Hospital of Traditional Chinese Medicine, Yueyang, China; ^3^ Yunnan Provincial Health Commission, Kunming, China; ^4^ Institute of Medical History and Literature, Chinese Academy of Chinese Medicine, Beijing, China; ^5^ Yunnan Provincial Hospital of Traditional Chinese Medicine, Kunming, China, yn-tcm-hospital.com

**Keywords:** acupuncture, bone metastatic cancer pain, network meta-analysis, TSA

## Abstract

**Background:**

Cancer pain (CP) is predominantly caused by bone metastases, imposing a significant burden on both patients and society. The limitations of the three‐step analgesic (TSA) therapy in managing CP have become increasingly evident. Although acupuncture demonstrates promising therapeutic effects for this condition, there is currently limited research comparing the efficacy of different acupuncture modalities.

**Results:**

A total of 20 studies were included, involving 1640 patients. The tumor types included lung cancer, prostate cancer, colorectal cancer, bile duct cancer, and breast cancer. A total of 9 acupuncture therapies were involved: acupoint embedding, auricular point pressing, electroacupuncture, ordinary acupuncture, bee therapy, fire needle therapy, moxibustion, acupoint injection, and acupoint application. The main outcome indicators were pain relief rate and NRS score, while the secondary outcome was the incidence of adverse reactions. The consistency test results showed no significant inconsistency among the comparison groups (*p* > 0.05), and a consistency model analysis was adopted. In terms of pain relief rate, electroacupuncture combined with auricular point pressing combined with the TSA therapy ranked the top three (SUCRA = 0.90), moxibustion combined with the TSA therapy ranked the top three (SUCRA = 0.84), and auricular point pressing combined with the TSA therapy ranked the top three (SUCRA = 0.65). In terms of reducing NRS scores, auricular point pressing combined with the TSA therapy ranked the top (SUCRA = 0.95), bee therapy combined with the TSA therapy ranked the top (SUCRA = 0.83), and acupuncture combined with acupoint injection combined with the TSA therapy ranked the top (SUCRA = 0.70). In terms of safety, the adverse reaction rate of fire needle therapy combined with the TSA therapy was the lowest (SUCRA = 0.98), electroacupuncture combined with auricular point pressing combined with the TSA therapy ranked the top (SUCRA = 0.81), and acupoint embedding combined with the TSA therapy ranked the top (SUCRA = 0.67). In terms of overall efficacy and safety, auricular point pressing combined with the TSA therapy performed the best in the benefit‐risk assessment.

**Conclusion:**

Acupuncture combined with the TSA therapy can effectively relieve CP in bone metastasis, with fewer adverse reactions. The current evidence indicates that auricular point pressing combined with the TSA therapy is the best treatment option in terms of comprehensive benefits. Due to the limited number of included studies, low quality of evidence, and potential bias risks, the conclusion still needs to be further verified by high‐quality research.

## 1. Introduction

Cancer pain (CP) is one of the most common symptoms in patients with advanced cancer, most of which are caused by bone metastases [1]. It is particularly common in solid tumors such as breast cancer, prostate cancer, and lung cancer, where bone metastases in breast cancer patients can be as high as 65%–75% [2]. As cancer patients live longer, the incidence of bone metastases is on the rise, and about 75% of patients with bone metastases experience varying degrees of pain [3]. This pain not only seriously affects the quality of life of patients but is also closely associated with a series of bone‐related events, including pathological fractures, spinal cord compression, and hypercalcemia [4]. Bone metastatic CP imposes a heavy burden of disease on patients and society. It not only severely affects patients’ ability to perform daily activities and sleep quality but may also lead to psychological problems such as depression and anxiety. From the perspective of healthcare resource utilization, patients with bone metastases require frequent imaging examinations, pain management, and treatment of complications, which places a huge financial burden on the medical system.

The most commonly used treatment currently is the three‐step analgesic therapy (TSA). Although TSA has been widely used in CP management, its effectiveness in clinical practice still faces many challenges. Based on pain intensity grading, this regimen progresses from nonopioids to weak opioids and then to strong opioids, aiming to provide individualized analgesia for patients. However, with the extension of treatment time, patients tend to develop drug tolerance, which leads to a gradual weakening of the analgesic effect and the need for increasing drug doses. Such dose increments may not only exacerbate side effects but also pose potential risks such as drug dependence. In addition, there are individual differences in response to opioids, with about 20–30 percent of patients not achieving satisfactory analgesic effects—a condition known as opioid‐refractory pain. In terms of side effects, opioids often cause gastrointestinal symptoms such as constipation, nausea, and vomiting, as well as central nervous system adverse reactions (AEs), including drowsiness and respiratory depression [5]. For patients with bone metastatic CP, due to the complex mechanism of pain involving multiple factors such as inflammation and neuropathic changes, relying solely on TSA often fails to achieve the desired analgesic effect [6]. At the same time, long‐term use of opioids may also suppress immune function and increase the risk of infection, which is particularly disadvantageous for cancer patients who are already in an immunosuppressed state. These limitations highlight the insufficiency of TSA in CP management, and there is an urgent need to explore safer and more effective alternative or complementary treatments.

Recently, a network meta‐analysis by Wang et al. [7] evaluated acupuncture combined with conventional therapy for cancer‐induced bone pain. However, their analysis did not include bee therapy, fire needle, or point application, nor did they assess the benefit‐risk balance or conduct meta‐regression on treatment frequency and duration. Our study extends this evidence by incorporating these additional modalities and analytical approaches. In the field of CP management, acupuncture is gaining increasing attention as a nonpharmacological intervention. The analgesic mechanisms of acupuncture involve multiple pathways: studies have shown that acupuncture can produce central analgesic effects by activating the endogenous opioid peptide system (e.g., β‐endorphin and enkephalins); it can also activate descending inhibitory pathways to suppress the transmission of pain signals at the spinal dorsal horn; additionally, acupuncture exerts anti‐inflammatory effects by inhibiting the release of inflammatory cytokines such as IL‐1β and TNF‐α, thereby alleviating inflammatory pain in the bone metastatic microenvironment [8, 9]. Existing studies suggest that acupuncture, as an adjunct treatment, has shown potential in alleviating bone metastatic CP. A meta‐analysis involving 1069 patients from 13 randomized controlled trials (RCTs) showed that acupuncture combined with conventional treatment significantly reduced CP intensity (MD = −1.34, 95% CI −1.74 to −0.94), decreased the frequency of breakthrough pain, shortened the onset time of analgesia, prolonged the duration of analgesia, and improved patients’ quality of life [8]. Another meta‐analysis showed that acupuncture combined with TSA had better analgesic effects on bone metastatic CP and reduced the incidence of AEs [10]. However, existing studies have only compared acupuncture plus TSA versus TSA alone, without systematically comparing the relative efficacy of different acupuncture modalities combined with TSA. Different types of acupuncture (e.g., manual acupuncture, electropuncture, fire needle, auricular point sticking, acupoint catgut embedding, acupoint injection, moxibustion, apitherapy, and point application) differ significantly in their operational approach, stimulation intensity, and therapeutic targets, and their efficacy and safety for bone metastatic CP may also vary. Traditional pairwise meta‐analysis can only perform direct comparisons between two interventions and cannot simultaneously rank multiple treatment options. Network meta‐analysis, by contrast, integrates both direct and indirect evidence to evaluate the comparative efficacy of multiple interventions even in the absence of head‐to‐head trials, thereby providing more comprehensive evidence to inform clinical decision‐making. Therefore, conducting a network meta‐analysis of different acupuncture modalities combined with TSA for bone metastatic CP is of significant clinical importance and methodological necessity. This study systematically searched for RCTs and employed network meta‐analysis to compare the pain relief rate (PRE), NRS score, and AE incidence of nine different acupuncture modalities combined with TSA, and ranked them to provide evidence‐based guidance for optimizing clinical acupuncture selection.

## 2. Methods

### 2.1. Registration

This study was conducted in accordance with PRISMA requirements. We have registered this protocol with PROSPERO (ID: CRD420251078017).

### 2.2. Inclusion Criteria

Population: Patients diagnosed with malignant tumor, with bone metastasis confirmed by imaging, accompanied by bone metastasis symptoms such as bone pain, pathological fractures, movement disorders, spinal nerve and spinal cord compression, and obvious pain location. Tumor type, age, gender, nationality, etc. are not restricted.

Intervention: Acupuncture, electropuncture, fire needle, auricular point sticking, acupoint injection, point application, etc., combined with TSA therapy.

Comparison: TSA therapy.

Outcome: Primary outcomes: [1] PRE, defined as the number of patients with pain relief divided by the total number of patients × 100%. Pain was assessed using the Visual Analog Scale (VAS), ranging from 0 to 10. Evaluation criteria: complete remission (CR) = VAS reduced to 0; marked relief (PR) = VAS reduced by ≥ 1/2 and sleep/normal life not affected; mild relief (MR) = VAS reduced by approximately 1/2 but sleep affected; no effect (NP) = no significant change in VAS [10]. We conducted a review of all the studies included in the research, confirming that the definitions of PRE in each study were basically the same, all adopting the above four‐category criteria (CR, PR, MR, and NP), and calculating the PRE as (CR + PR + MR)/total number of cases × 100%. No studies were found to use different classification thresholds or calculation methods. This definition originated from literature [10] and is a widely used efficacy evaluation standard in CP research. Therefore, there is no need for cross‐study definition correction or statistical adjustment. The relief standard in the PRE definition (a 50% reduction in VAS or reaching 0) is consistent with the clinical significant relief threshold recommended by the NCCN Adult CP Guidelines (a 30% reduction in VAS for partial relief and a 50% reduction for significant relief). Among them, a 50% reduction in VAS (50%) is widely regarded as a clinically significant response threshold in CP clinical trials. Therefore, the PRE definition adopted in this study conforms to the clinical validation standard [2]. Numerical Rating Scale (NRS) score [11], graded from 0 to 10 (0 = painless; 1–3 = mild pain not affecting sleep or daily life; 4–6 = moderate pain affecting sleep and life; 7–10 = severe pain, intolerable).

Secondary outcome: AEs.

Study design: RCT.

### 2.3. Exclusion Criteria

Interventions not met; patients with nonbone metastatic CP; reviews, animal experiments, abstracts, letters, etc. Duplicate literature; no access to the original text; the data are incomplete or cannot be extracted.

### 2.4. Data Sources and Search Strategy

RCTs of acupuncture for CP caused by bone metastases published in PubMed, EMbase, Cochrane Library, Web of Science, CNKI, CSCD, CCD, and CBM were retrieved until July 1, 2025 (Supporting Information 1. PubMed retrieval strategy).

### 2.5. Study Selection

Two researchers imported the search records from each database into the literature management software Endnote X9, and two researchers independently screened and extracted the relevant data of the literature that met the inclusion criteria. Cross‐check the included literature, and in case of any discrepancy, decide whether to include it through discussion or by a third, more experienced researcher.

### 2.6. Data Extraction

Two researchers designed data extraction tables based on the information needed for the study and extracted them independently. The contents included ① Basic information: title, author, year, study type, diagnostic criteria, intervention measures, course of treatment, and outcome indicators. ② Demographic characteristics: sample size, age, gender. ③ Methodological information: random method, allocation hide scheme, blinding, etc. When the information extracted by the two people is inconsistent, they first discuss and resolve it with each other. If it still cannot be resolved, they discuss and decide.

### 2.7. Risk of Bias Instudies

Two researchers (Zhao BF and Wang SH) used the Cochrane Randomized Controlled Trials Risk of Bias assessment tool [12] to assess the risk of bias. The assessment tool included the following seven items: generation of random sequences, allocation concealment, blinding of subjects and intervention providers, blinding of outcome evaluators, incomplete outcome data, selective outcome reporting, and other sources of bias. Each item was assessed as low bias, high bias, or unclear.

### 2.8. Transitivity Assumption

The validity of the network meta‐analysis depends on the assumption of transmissibility, which means that the distribution of effect modifiers among different comparisons should be similar. We evaluated the transmissibility by comparing the distributions of the following clinical and methodological variables across studies: average age, gender distribution, tumor type, treatment frequency, treatment course, and the operational characteristics of different acupuncture therapies. Considering that there are certain differences in acupuncture patterns, stimulation intensity, treatment frequency, and course among the studies, we conducted the following additional analyses to assess the transmissibility: (1) We tested the moderating effect of treatment frequency and course on the efficacy through Meta regression analysis; (2) we conducted descriptive subgroup comparisons by acupuncture patterns to evaluate the consistency of effect trends among different patterns; (3) we marked the treatment frequency and course of each study in the evidence network diagram to observe whether there is a systematic pattern. The results showed: (1) Meta regression analysis did not find an interaction between treatment frequency and course and the intervention measures (*p* > 0.05), suggesting that these variables are not significant effect modifiers; (2) different acupuncture patterns all showed a trend of being superior to simple TSA in reducing NRS scores, and the effect directions were consistent; and (3) baseline pain intensity, age, gender, and tumor type were distributed relatively evenly among the comparisons. Therefore, the assumption of transmissibility is considered reasonable. However, due to the lack of head‐to‐head direct comparisons among different acupuncture patterns, we still need to interpret the ranking results with caution and acknowledge this limitation in the discussion.

### 2.9. Statistical Analysis

Network meta‐analysis was conducted using R Version 4.2.0 with the netmeta package (version 2.1–0). A random‐effects model was used for all analyses due to anticipated clinical and methodological heterogeneity across the included studies. For binary variables, relative risk (RR) was used; for continuous variables, mean difference (MD) was used; and each effect size was reported with a 95% confidence interval (CI). The I^2^ statistic was used to assess the magnitude of heterogeneity: I^2^ ≤ 50% indicated no statistical heterogeneity, while I^2^ > 50% indicated statistical heterogeneity, in which case sensitivity analysis was conducted to explore sources of heterogeneity. Network evidence plots were generated for each outcome measure. When closed loops appeared in the network, an inconsistency test was performed. If *p* > 0.05, the consistency model was used for analysis; if *p* < 0.05, inconsistency was reported and the node‐splitting method was used for inconsistency testing. For each outcome, interventions were ranked using the surface under the cumulative ranking curve (SUCRA), expressed as a percentage (higher percentage indicates better intervention). When the number of included studies for an outcome was ≥ 10, a comparison‐adjusted funnel plot was generated and publication bias tests were performed. Sensitivity analysis was used to test the stability of the results. Meta‐regression was used to explore the influence of different factors (e.g., treatment frequency and duration) on the results.

### 2.10. Evaluation of Evidence Quality

We used CINeMA [13] to assess the quality of evidence. CINeMA graded the quality of evidence in six areas, namely, intrastudy bias, interstudy bias, indirectness, imprecision, heterogeneity, and inconsistency, and the final quality of evidence was classified as high, medium, low, and very low quality grades.

## 3. Results

### 3.1. Search Results

**FIGURE 1:**
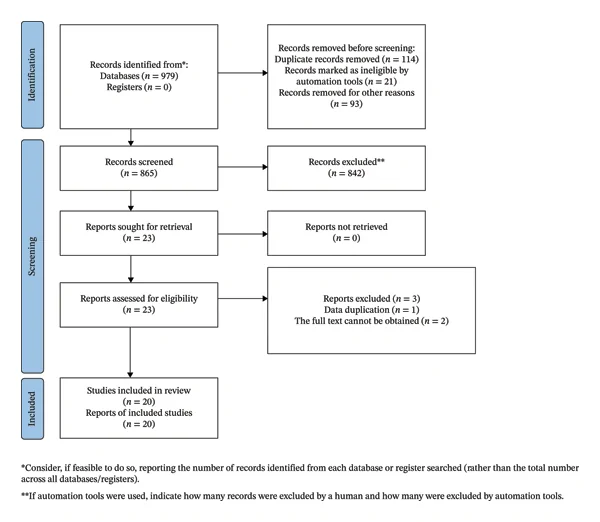
Literature screening flowchart (PRISMA flowchart).

A preliminary search in both Chinese and English databases yielded 979 studies, while no matching studies were retrieved through other means. After checking for duplicates, 865 documents were obtained. A total of 842 studies were obtained by reading the titles and abstracts, and 3 were excluded by reading the full text, resulting in 20 studies [14–32] (Figure 1).

### 3.2. Basic Characteristics of the Included Literature

A total of 1640 cases were included in 20 studies for statistical analysis, including 823 cases in the intervention group and 817 cases in the control group. Tumor types included lung cancer, prostate cancer, intestinal cancer, cholangiocarcinoma, breast cancer, etc. There are nine acupuncture methods: acupoint catgut embedding, auricular point sticking, electropuncture, acupuncture, apitherapy, acupoint injection and fire needle, moxibustion, and point application (Table 1).

**TABLE 1:** Basic characteristics of the included literature.

Researchers	Year	Sample size		Cancer type	Age		Gender (male: female)		Interventions		Frequency	Course	Outcomes
		Experimental group	Control group		Experimental group	Control group	Experimental group	Control group	Experimental group	Control group			
Jiang [13]	2014	30	30		60.3 ± 3.36	61.47 ± 3.21	17:13	16:14	2 and 1	1	Once every 7 days	7 days	PRE, NRS, AE
Wang et al. [14]	2015	30	30	Multitype	62.87 ± 10.96		23:37		3 and 1	1	Three times a day		PRE, NRS, AE
Wu et al. [15]	2017	58	56	Multitype	63.10 ± 6.88	61.70 ± 7.19	32:26	32:24	4 and 3 and 1	1	Twice a day	21 days	PRE
Chu et al. [16]	2018	30	29	Multiple types	61.01 ± 9.81	60.09 ± 9.78	15:15	13:16	5 and 1	1	Once a day	7 days	PRE, NRS, AE
Wu et al. [17]	2018	26	26	Multitype			13:13	11:15	6 and 1	1	Once a day	28 days	NRS
Lu et al. [18]	2018	30	30		62.50 ± 10.06	62.50 ± 10.06	14:16	15:15	5 and 1	1	Twice a day	5 days	PRE, NRS
Liu et al. [19]	2019	43	40	Lung cancer	63.92 ± 8.47	64.08 ± 8.52	28:15	26:14	5 and 1	1	Once a day	21 days	PRE, NRS, AE
Wu et al. [20]	2020	80	80	Lung cancer	59.28 ± 3.36	58.13 ± 3.46	52:28	53:27	5 and 7 and 1	1	Twice a day	28 days	PRE, NRS, AE
Yu [21]	2020	22	22	Lung cancer			14:8	13:9	5 and 1	1	Once a day	7 days	NRS, AE
Chen et al. [22]	2020	30	30	Lung cancer					5 and 1	1	Once a day	28 days	PRE, AE
Gou et al. [23]	2021	40	40	Multitypes	61.45 ± 16.28	61.62 Earth 16.11	21:19	22:18	2 and 1	1	Once every 7 days	14 days	PRE, AE
Yan [24]	2021	60	60	Multiple types	59.72 ± 15.87	60.44 ± 15.62	36:24	38:22	5 and 1	1	Once every 5 days	15 days	PRE, NPS
Ni et al. [25]	2021	40	40	Lung cancer	56.28 ± 7.10	53.88 ± 6.23	30:10	33:7	5and3and1	1	Three times a day	14 days	PRE, NRS, AE
Zhang [26]	2022	30	30	Multitypes	57 ± 10	55 ± 9	16:14	15:15	4 and 3 and 1	1	Once every 7 days	7 days	PRE, NRS, AE
Liu [27]	2023	30	30	Multitype	58.40 ± 12.22	60.30 ± 9.56	17:13	16:14	5 and 1	1	Once every 7 days	28 days	PRE, NRS, AE
Yan [28]	2024	42	42	Prostate cancer	73.14 ± 5.07	73.98 ± 6.78	42:0	42:0	3 and 1	1	Five times a day	56 days	PRE
Zhang et al. [29]	2024	100	100		54.54 ± 8.23	54.22 ± 8.54	54:46	63:37	8	1	Twice a day	14 days	PRE, AE
Zhu [30]	2024	30	30	Multitypes	44.52 ± 5.20	4.36 ± 5.12	15:15	14:16	9 and 1	1	Once every 7 days	10 days	PRE
Li [31]	2025	32	32	Multitype	47.69 ± 7.15	47.75 ± 7.16	18:14	20:12	5 and 1	1	Once every 7 days	20 days	PRE, AE
Liang [32]	2025	40	40	Multiple types	57.82 ± 12.67	55.50 ± 11.65	23:17	26:14	10 and 1	1	Once every 7 days	7 days	PRE, NRS, AE

*Note:* 1: Three‐step analgesia therapy; 2: acupoint catgut embedding; 3: auricular point sticking; 4: electroacupuncture; 5: acupuncture; 6: apitherapy; 7: acupoint injection; 8: fire needle; 9: moxibustion; 10: point application. Adverse reactions (AE).

Abbreviations: NRS, Numerical Rating Scale; PRE, pain relief rate.

### 3.3. Quality Evaluation

**FIGURE 2:**
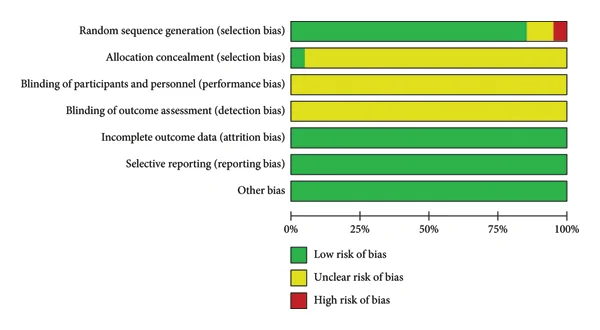
Risk of bias assessment results of included studies (ROB2).

**FIGURE 3:**
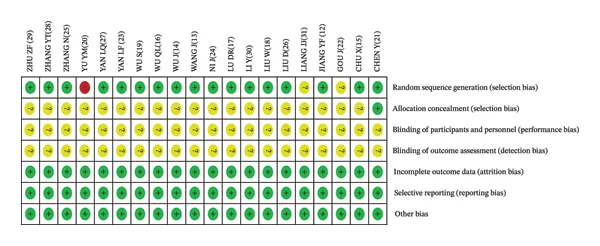
Summary of risk of bias assessment results of included studies (ROB2).

In terms of random sequence generation, 18 studies (90%) used random number tables or computer software to generate random sequences and were rated as having low bias risk; 1 study used the order of visits for allocation and was rated as having high bias risk; 1 study did not describe the random method and was rated as unclear. In terms of allocation concealment, only 1 study (5%) clearly used the envelope method for allocation concealment and was rated as having low bias risk; the remaining 19 studies (95%) did not report the allocation concealment scheme and were rated as unclear. In terms of blinding, due to the special nature of acupuncture intervention, neither the subjects nor the intervention providers could implement blinding, and all 20 studies (100%) were rated as having high bias risk in this area; in terms of outcome assessment blinding, all studies did not clearly report, and were rated as unclear. Regarding incomplete outcome data, all 20 studies (100%) fully reported the outcome data without any loss to follow‐up or data missing and were rated as having low bias risk. Regarding the assessment of selective reporting bias, we judged by comparing the outcome indicators mentioned in the original studies with the preset outcome indicators in the methodology section. If the study did not report the preset important outcomes (such as AEs and NRS scores), it was determined to have selective reporting bias. The assessment results showed that all 20 studies fully reported the preset outcome indicators, and the selective reporting bias was low risk. Regarding the registration of the trial protocol, we checked whether each included study clearly mentioned the trial registration number and registration platform. The results showed that the lack of protocol registration might increase the risk of selective reporting bias, but based on the completeness assessment of outcome reports in published literature, all studies reported the main outcome indicators. Regarding the handling of missing data, we extracted the methods for handling missing data from each study. Among the 20 included studies, all studies reported no missing data or cases of loss to follow‐up, and the outcome data integrity was rated as low risk. In other biases, no obvious other sources of bias were found, and all 20 studies (100%) were rated as having low bias risk. Overall, the included studies had good quality in terms of outcome data completeness and selective reporting, but the allocation concealment report was insufficient, and blinding was difficult to implement, which are the inherent limitations of acupuncture clinical research (Figures 2 and 3).

### 3.4. Meta‐Analysis

#### 3.4.1. PRE

**FIGURE 4:**
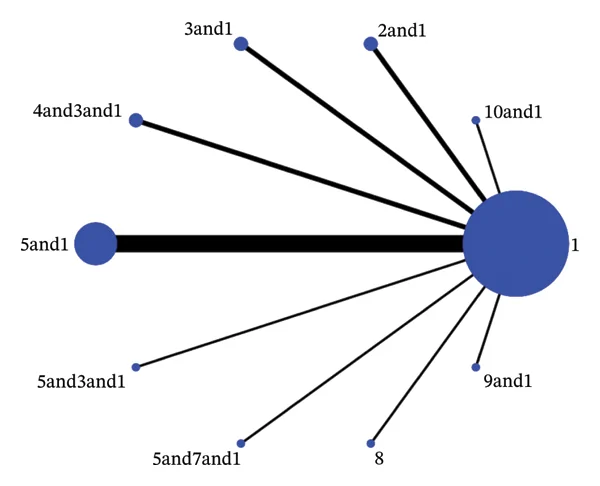
Network evidence for PRE.

**FIGURE 5:**
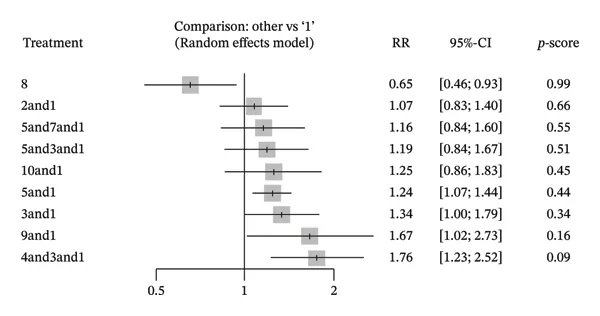
Forest plot of network meta‐analysis for PRE.

A total of 18 studies reported the PRE. The evidence network contained no closed loops (Figure 4). As no closed loops were present in the evidence network, formal inconsistency testing could not be performed. All comparisons were therefore based on indirect evidence through the common TSA comparator. Heterogeneity testing showed I^2^ = 12%, suggesting low heterogeneity. The meta‐analysis results showed that compared with TSA alone, electroacupuncture combined with auricular point pressing combined with TSA, moxibustion combined with TSA, auricular point pressing combined with TSA, and acupuncture combined with TSA had significant advantages in improving the PRE (*p* < 0.05) (Figure 5, Supporting Information 2). The SUCRA rankings were as follows: electroacupuncture combined with auricular point pressing combined with TSA (0.90), moxibustion combined with TSA (0.84), and auricular point pressing combined with TSA (0.65) (Table 2).

**TABLE 2:** SUCRA sorting.

Intervention	Pain relief rate		NRS		Adverse reactions	
	SUCRA	No	SUCRA	No	SUCRA	No
2 and 1	0.33	8	0.23	7	0.67	3
3 and 1	0.65	3	0.92	1	0.53	4
4 and 3 and 1	0.90	1	0.21	8	0.81	2
5 and 3 and 1	0.48	6	0.40	6	0.49	5
5 and 1	0.56	4	0.61	4	0.45	6
6 and 1	—	—	0.83	2	—	—
5 and 7 and 1	0.44	7	0.70	3	0.06	9
9 and 1	0.84	2	—	—	—	—
10 and 1	0.55	5	0.46	5	0.36	7
8	0.005	10	—	—	0.98	1
1	0.20	9	0.09	9	0.10	8

*Note:* 1: Three‐step analgesia therapy; 2: acupoint catgut embedding; 3: auricular point sticking; 4: electroacupuncture; 5: acupuncture; 6: apitherapy; 7: acupoint injection; 8: fire needle; 9: moxibustion; 10: point application.

#### 3.4.2. NRS

**FIGURE 6:**
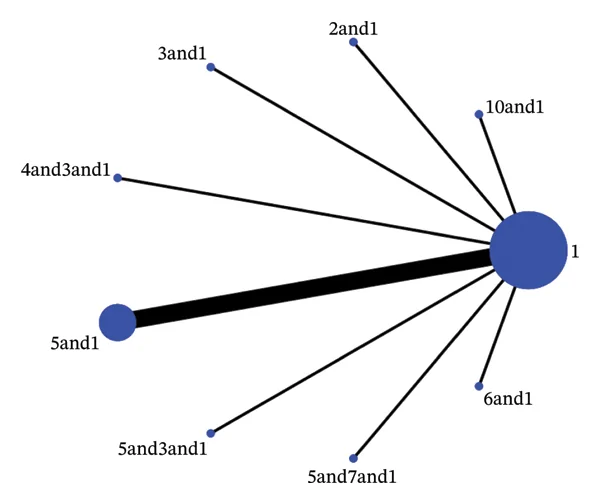
Network evidence for NRS.

**FIGURE 7:**
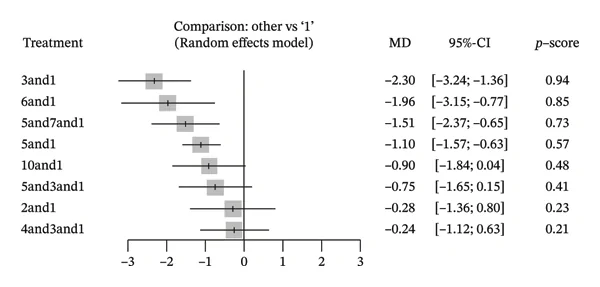
Forest plot of network meta‐analysis for NRS.

A total of 12 studies reported the NRS scores. The evidence network contained no closed loops (Figure 6). As no closed loops were present in the evidence network, formal inconsistency testing could not be performed. All comparisons were therefore based on indirect evidence through the common TSA comparator. Heterogeneity testing showed I^2^ = 14%, indicating low heterogeneity. The meta‐analysis results showed that compared with TSA alone, auricular point pressing combined with TSA, bee therapy combined with TSA, acupuncture combined with acupoint injection combined with TSA, and acupuncture combined with TSA had significant advantages in reducing NRS scores (*p* < 0.05) (Figure 7, Supporting Information 2). The SUCRA rankings were as follows: auricular point pressing combined with TSA (0.92), apitherapy combined with TSA (0.83), and acupuncture combined with acupoint injection combined with TSA (0.70) (Table 2).

#### 3.4.3. AEs

**FIGURE 8:**
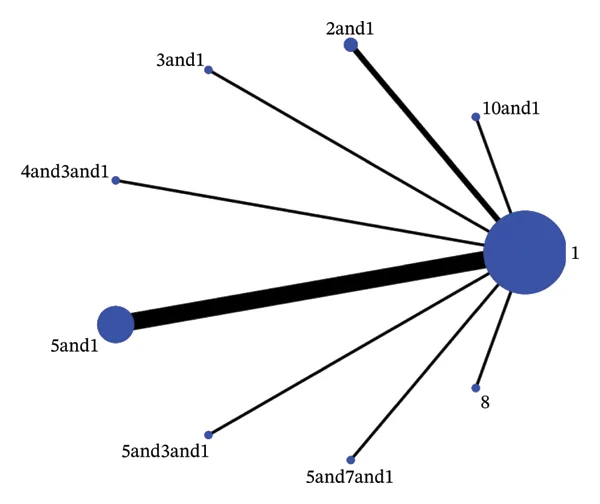
Network evidence for AE.

**FIGURE 9:**
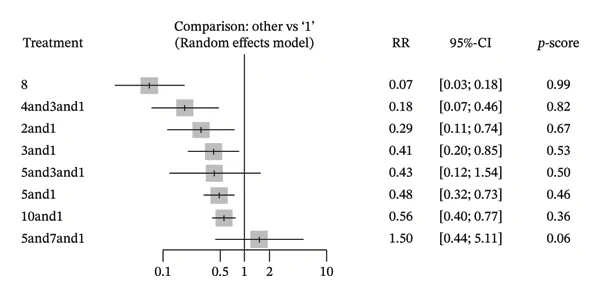
Forest plot of network meta‐analysis for AE.

A total of 14 studies reported the incidence of AEs. The evidence network contained no closed loops (Figure 8). As no closed loops were present in the evidence network, formal inconsistency testing could not be performed. All comparisons were therefore based on indirect evidence through the common TSA comparator. Heterogeneity testing showed I^2^ = 2%, indicating extremely low heterogeneity. The meta‐analysis results showed that compared with TSA alone, the incidence of AEs was significantly lower in the fire needle, electroacupuncture combined with auricular point pressing combined with TSA, acupoint embedding combined with TSA, auricular point pressing combined with TSA, acupuncture combined with auricular point pressing combined with TSA, acupuncture combined with TSA, and acupoint application combined with TSA (*p* < 0.05) (Figure 9, Supporting Information 2). The SUCRA rankings were as follows: fire needle combined with TSA (0.98), electroacupuncture combined with auricular point pressing combined with TSA (0.81), and acupoint embedding combined with TSA (0.67) (Table 2).

#### 3.4.4. Comprehensive Assessment of Benefits and Risks

Based on the SUCRA ranking of overall efficacy and safety, although fire needling performed the best in terms of safety (SUCRA = 0.98), its analgesic effect was relatively limited; electroacupuncture combined with auricular point pressing ranked first in terms of PRE (SUCRA = 0.90), but the operation was more complex; auricular point pressing combined with the TSA therapy ranked first in reducing the NRS score (SUCRA = 0.95), ranked third in PRE (SUCRA = 0.65), had good safety, was simple to operate, and had the optimal overall benefit‐risk assessment. In conclusion, based on the current limited evidence, auricular point pressing combined with the TSA therapy may be a promising acupuncture combination protocol, but these findings should be regarded as exploratory evidence rather than confirmatory conclusions.

#### 3.4.5. Occurrence of AEs

The AEs included in the study mainly included nausea, vomiting, constipation, dizziness, etc. Table 3 shows the occurrence of AEs.

**TABLE 3:** Adverse reactions occur.

Researchers	Symptom	
	Experimental group	Control group
Jiang YF	1 case of nausea and vomiting, 2 cases of loss of appetite and 3 cases of constipation	5 cases of dyspnea, 8 cases of nausea and vomiting, 8 cases of loss of appetite, 14 cases of constipation
Wang J	4 cases of nausea and vomiting, 3 cases of constipation	3 cases of nausea and vomiting, 14 cases of constipation
Chu X	Five cases of nausea and vomiting, three cases of dizziness, one case of constipation, two cases of drowsiness, and one case of subcutaneous bleeding	Nine cases of nausea and vomiting, four cases of dizziness, three cases of constipation, and four cases of drowsiness
Liu W	1 case of constipation, 1 case of dizziness, 1 case of subcutaneous bleeding	4 cases of constipation, 2 cases of dizziness, 1 case of nausea and vomiting, 2 cases of drowsiness
Wu S	There were 2 cases of nausea and vomiting, 3 cases of pruritus, and 1 case of constipation	Two cases of nausea and vomiting, one case of pruritus, and one case of constipation
Yu YM	1 case of nausea, 1 case of constipation, 2 cases of subcutaneous bleeding	2 cases of nausea, 4 cases of constipation, 1 case of pruritus, 1 case of drowsiness
Chen Y	1 case of nausea and vomiting, 1 case of constipation	Nausea and vomiting 2, dizziness 2, constipation 3, subcutaneous bleeding 1, drowsiness 1
Gou J	1 case of fracture, 1 case of spinal cord compression, 2 cases of hypercalcemia	2 fractures, 4 spinal cord compression, 5 hypercalcemia
Ni J	1 case of nausea, 1 case of dizziness, 1 case of urticaria	2 cases of constipation, 2 cases of nausea, 2 cases of dizziness, 1 case of rash
Zhang N	4 cases of nausea	There were 8 cases of nausea and 5 cases of constipation
Liu DL	Three cases of nausea and vomiting, three cases of constipation and one case of drowsiness	5 cases of nausea and vomiting, 1 case of dizziness, 6 cases of constipation, 1 case of drowsiness
Zhang YT	2 cases of nausea and 2 cases of dizziness	18 cases of nausea, 12 cases of dizziness, 25 cases of constipation, 4 cases of fever
Ly Y	1 case of nausea, 1 case of dizziness, 1 case of constipation	2 cases of nausea and vomiting, 1 case of constipation, 1 case of dizziness
Liang JJ	3 cases of nausea and vomiting, 3 cases of dizziness, 10 cases of constipation, 3 cases of loss of appetite, 1 case of difficulty in urination	8 cases of nausea and vomiting, 5 cases of dizziness, 18 cases of constipation, 4 cases of loss of appetite and 1 case of difficulty in urination

#### 3.4.6. Publish Bias Analysis

**FIGURE 10:**
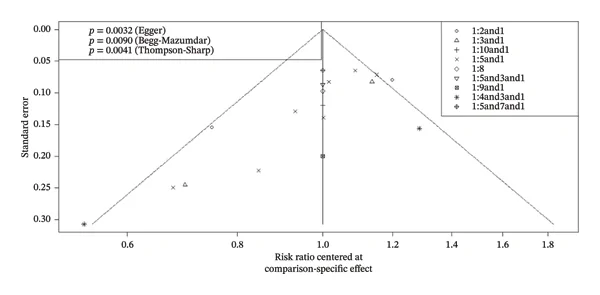
Publication bias funnel plots for PRE.

**FIGURE 11:**
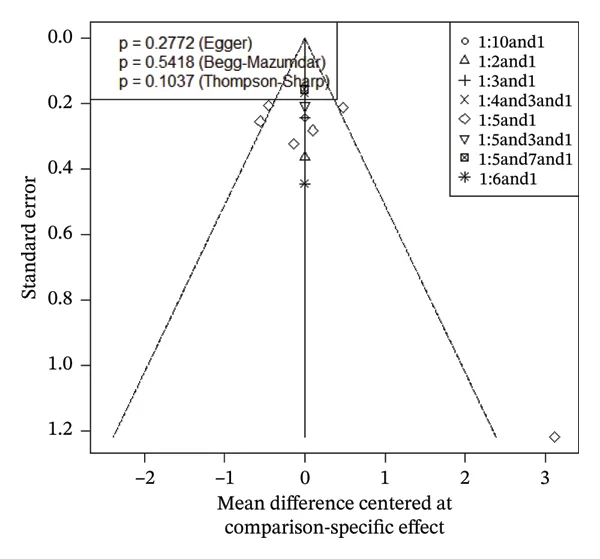
Publication bias funnel plots for NRS.

**FIGURE 12:**
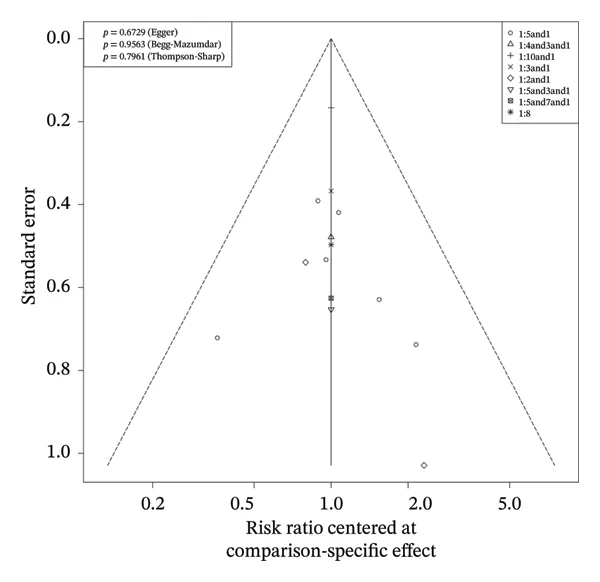
Publication bias funnel plots for AE.

Publication bias was detected for the PRE through the funnel plot and publication bias tests (Figures 10, 11, 12). To address this, we performed a trim‐and‐fill analysis. The analysis imputed 3 potentially missing studies. After adjustment, the pooled RR changed from 1.242 (95% CI: 1.096–1.407) before adjustment to 1.097 (95% CI: 0.955–1.259), and the CI crossed 1, indicating that the adjusted effect was no longer statistically significant. This suggests that the observed analgesic effect for PRE may be overestimated due to publication bias. Therefore, we have substantially downgraded the confidence in the PRE‐related conclusions. These findings should be interpreted with caution, and the results for PRE are presented as exploratory rather than confirmatory evidence.

#### 3.4.7. Sensitivity Analysis and Meta‐Regression

We performed sensitivity analysis using the leave‐one‐out method. The results showed that the combined effect sizes and directions for each outcome did not change significantly after removing any single study, indicating good stability of the results (Supporting Information 3).

Meta‐regression was performed on treatment frequency and treatment duration to explore the influence of these factors on efficacy. The results indicated that compared with the control group, treatment frequency and duration were significantly correlated with efficacy. For NRS scores, the effect of acupuncture combined with TSA was proportional to treatment frequency and duration. For AEs, the effect of acupuncture combined with TSA was inversely proportional to treatment frequency and duration. The effect of point application combined with TSA was inversely proportional to treatment frequency, and the effect of fire needle was inversely proportional to treatment duration (Table 4).

**TABLE 4:** Meta‐regression results.

Outcome indicator	Intervening measure	Frequency (RR/MD [95% CI])	Course (RR/MD [95% CI])
NRS	1vs5and1	−1.16 (−2.37, −0.50)	−1.35 (−2.79, −0.30)
Adverse reaction	1vs10and1	−2.30 (−4.29, −0.39)	
	1vs5and1	−1.04 (−2.02, −0.10)	−1.02 (−1.96,‐0.09)
	1vs8		−3.64 (−6.92,‐0.48)

*Notes:* 1: Three‐step analgesia therapy; 5: acupuncture; 8: fire needle; 10: point application.

#### 3.4.8. Evaluation of Evidence Quality

The CINeMA results for each acupuncture method compared with conventional treatment showed generally low quality of evidence, with downgrading mainly due to selective reporting and imprecision (Supporting Information 4). In addition, due to the detection of publication bias for PRE, we have further downgraded the confidence in the PRE‐related conclusions. This is explicitly noted in the Discussion and Conclusion sections.

## 4. Discussion

### 4.1. Summary and Interpretation of Main Findings

The key interpretative finding of this network meta‐analysis is that while most acupuncture‐TSA combinations show potential benefits for bone metastatic CP, the optimal choice depends on clinical priority. Auricular point sticking combined with TSA achieved the best overall balance, ranking first in NRS reduction (SUCRA = 0.95) and third in PRE (SUCRA = 0.65), with a favorable safety profile. Fire needle showed the highest safety ranking (SUCRA = 0.98) but modest analgesic efficacy. This discrepancy may be explained by fire needle being a minimally invasive technique with short‐lasting effects and its safety advantage being relative to opioid‐related systemic side effects rather than indicating an absence of local discomfort. Electroacupuncture combined with auricular point sticking ranked highest in PRE (SUCRA = 0.90) but involves complex procedures. AEs (nausea, vomiting, constipation, and dizziness) were primarily attributable to opioid use. However, SUCRA rankings alone should not be interpreted as definitive evidence of superiority, given the low to very low quality of evidence, overlapping CIs, and absence of head‐to‐head comparisons.

Before interpreting these rankings, we assessed the key assumptions underlying our network meta‐analysis. First, regarding transitivity, clinical and methodological variables were broadly similar across comparisons, supporting the transitivity assumption. Second, an important structural limitation is the absence of closed loops in the evidence network, meaning all comparisons were based on indirect evidence through TSA alone, which precludes formal inconsistency testing.

The included acupuncture modalities differed in operation methods, stimulation intensity, treatment frequency, and duration, which are potential sources of clinical heterogeneity. To assess the impact on transitivity, we conducted a multidimensional evaluation. First, all interventions shared TSA as a common comparator, with consistent TSA protocols across studies, providing an anchor for indirect comparisons. Second, meta‐regression showed that treatment frequency and duration were correlated with efficacy (positive for NRS, negative for AEs), but no interaction was found between these variables and intervention type (*p* > 0.05), indicating they are not significant effect modifiers. Third, despite differences in operation, all acupuncture modalities share common mechanistic pathways (e.g., endogenous opioid peptide activation and anti‐inflammatory effects), making indirect comparison clinically reasonable. However, due to the lack of head‐to‐head comparisons, formal inconsistency testing could not be performed, and residual confounding may still exist. Therefore, our efficacy rankings should be considered exploratory, and future head‐to‐head RCTs are needed for validation.

### 4.2. Mechanistic Rationale for Auricular Point Sticking

Auricular point sticking operates under the guidance of traditional Chinese medicine (TCM) meridian theory, leveraging the regulatory functions of meridians and acupoints on bodily systems. Clinically, it is used to diagnose and treat diseases based on the close relationship between the ear and internal organs via meridian pathways. Auricular point pressing is a therapeutic technique rooted in TCM meridian theory and biological holography. It involves applying *Plantago asiatica* seeds to specific auricular points, inducing local sensations such as soreness, numbness, distension, and pain. Through neural and meridian sensory transmission, it modulates organ function and qi‐blood circulation. From a modern anatomical perspective, the auricle contains a dense network of nerves and blood vessels, including somatic, sympathetic, and vagus nerves, which interweave to form a complex plexus. There is bidirectional information exchange between auricular acupoints and corresponding body regions. When an organ experiences pain, this information is transmitted to the auricle via a holographic reflex, triggering localized responses. Stimulating specific auricular points can thus produce analgesic effects. Moreover, auricular analgesia is cost‐effective, simple to administer, nonaddictive, minimally invasive, associated with few side effects, and has no time restrictions [33, 34]. Studies have shown that auricular point sticking as an adjunct to three‐step analgesia in patients with advanced prostate cancer and bone metastasis‐related pain not only alleviates pain but also reduces serum levels of pain mediators. Its mechanism may be closely related to its ability to regulate the secretion and release of these pain factors [29]. Auricular point stimulation can be integrated with the TSA protocol to manage CP, enabling dose reduction, mitigating drug‐related adverse effects, and exerting antiemetic and bowel‐regulating effects. Additionally, it modulates the central nervous system’s pain threshold, contributing to sedative, antispasmodic, and analgesic outcomes [15]. Clinical trials have reported high efficacy rates, with auricular acupuncture achieving a total effective rate of 95.1% in liver cancer patients and 93.1% in gastric cancer patients [35, 36].

### 4.3. Trade‐offs Between Efficacy and Safety and Clinical Implications

Other studies have demonstrated the efficacy of acupuncture for bone metastatic CP. He et al. [37] reported that acupuncture significantly alleviated CP (MD = −1.38, 95% CI:−2.13,−0.64). Si et al. [9] found that acupuncture combined with TSA achieved higher analgesic effectiveness than TSA alone (RR = 1.13, 95% CI:1.07,1.19), with lower pain intensity (MD = −1.27, 95% CI:−1.58,−0.96) and fewer AEs (RR = 0.51, 95% CI:0.42, 0.63). Epstein et al. [38] confirmed that acupuncture improves quality of life in cancer patients. Our study extends these findings by providing comparative rankings of different acupuncture modalities, which traditional pairwise meta‐analyses cannot offer.

Sensitivity analysis using the leave‐one‐out method confirmed the stability of our results. Meta‐regression revealed that the “dose‐effect” relationship varies across outcomes: for NRS improvement, the effect of acupuncture combined with TSA was positively correlated with treatment frequency and duration; for AEs, the effect was inversely correlated. However, these trends are not universal: point application efficacy was inversely proportional to frequency, and fire needle efficacy was inversely proportional to duration. These findings provide quantitative evidence for individualized treatment planning—optimizing frequency and duration by modality rather than blindly increasing treatment intensity.

Our findings reveal an important trade‐off between efficacy and safety across acupuncture modalities. Noninvasive techniques (e.g., auricular point sticking and acupoint embedding) demonstrated favorable efficacy‐safety balance, with auricular point sticking ranking highest in NRS reduction (SUCRA = 0.95). These techniques are easy to administer, have no systemic side effects, and are suitable for long‐term use. Invasive techniques showed varying patterns: fire needle achieved the highest safety ranking (SUCRA = 0.98) but modest efficacy, as its adverse events are local and transient; electroacupuncture ranked highest in PRE (SUCRA = 0.90) but requires specialized equipment.Clinical applicability: For patients seeking simple, safe, long‐term management, noninvasive techniques—particularly auricular point sticking—are most suitable. For those requiring rapid relief, electroacupuncture or manual acupuncture may be add‐on options. Fire needle may be reserved for patients highly sensitive to opioid side effects.

### 4.4. Advantages and Limitations

The clinical significance of this study lies in providing new treatment options for patients with bone metastasis CP, particularly in the context of opioid use limitations. However, several limitations should be acknowledged. Firstly, clinical heterogeneity. Differences in research design, intervention measures, and outcome indicators may exist. Although we quantified the moderating effects of treatment frequency and duration via meta‐regression, due to limited literature and reporting quality, we could not conduct subgroup analysis by primary tumor types (e.g., lung, breast, and prostate cancer). The biological behaviors of different tumors may theoretically affect acupuncture response. However, given the common pathophysiological mechanisms of bone metastatic pain across cancer types, our findings may have good extrapolation potential. Nevertheless, more large‐sample, stratified RCTs are needed. Secondly, opioid dosage. Some studies [36] suggest acupuncture may reduce opioid dosage, but this was rarely reported in our included studies and could not be analyzed. Thirdly, publication bias and small‐study effects. Publication bias was detected for PRE. Trim‐and‐fill analysis imputed 3 potentially missing studies; the adjusted RR changed from 1.242 (95% CI: 1.096–1.407) to 1.097 (95% CI: 0.955–1.259), crossing 1, indicating the original effect may have been overestimated. Contributing factors include: small‐study effects (75% of studies had sample size < 100; smaller studies reported larger effect estimates: RR = 1.28 vs. 1.09 for larger studies), selective publication of positive results common in CAM research, and regional publication bias (all 20 studies originated from China, where acupuncture is widely accepted). To mitigate these concerns, we performed trim‐and‐fill analysis, downgraded confidence in PRE‐related conclusions, and acknowledged these limitations. PRE findings should be interpreted with caution and considered exploratory. Fourthly, regional and language bias. All 20 studies originated from China. This geographic concentration may introduce regional publication bias, as cultural context may influence both research direction and publication likelihood. Studies from other regions (e.g., Europe, North America, Japan, and Korea) or in other languages (e.g., Japanese, Korean, and German) may have been missed. Future international collaborative studies are needed to confirm generalizability. Fifthly, PRE as a composite outcome. Although all studies used consistent PRE definitions, composite outcomes based on VAS change categories may be less sensitive than continuous outcomes like NRS. The 50% VAS reduction threshold may not capture more subtle but still important pain improvements. To address this, we included NRS as a co‐primary outcome to complement PRE. Sixthly, lack of patient preference data. We could not directly assess patient preferences for different modalities (e.g., willingness to accept local discomfort for better pain relief). Future studies should incorporate patient‐reported outcome measures and preference assessments to inform shared decision‐making. Despite these limitations, this study provides supportive evidence for acupuncture in treating bone metastatic CP. Future large‐scale, rigorous RCTs are needed to further verify its efficacy and safety.

Therefore, despite the promising findings, future large‐scale, rigorous RCTs are essential to further confirm the efficacy and safety of acupuncture for bone metastatic CP. Several critical issues remain to be addressed. First, standardization of acupoint selection is a key concern. Current studies exhibit considerable variability in acupoint combinations (e.g., Zusanli ST36 and Hegu LI4), which may contribute to heterogeneity in treatment outcomes [8]. Second, optimization of treatment frequency requires further investigation. There is no consensus on standardized treatment intervals or course durations, which may influence cumulative therapeutic effects and long‐term sustainability. Third, identification of biomarkers represents an important direction. Investigating dynamic changes in inflammatory factors such as CXCL12 could provide objective indicators for evaluating acupuncture efficacy [39]. From a methodological perspective, improving study design remains crucial. Triple‐blind RCTs using sham acupuncture controls are vital for minimizing bias. Additionally, developing patient‐controlled percutaneous acupoint electrical stimulation techniques could enhance intervention consistency.

## 5. Conclusion

Acupuncture therapy combined with the TSA therapy demonstrates a favorable analgesic effect in managing bone metastatic CP, with a relatively low incidence of AEs. Emerging evidence suggests that auricular point sticking, when integrated with the TSA approach, may represent a promising intervention among various acupuncture modalities. However, due to the low to very low certainty of evidence across most comparisons, these findings must be interpreted with caution. Further high‐quality, rigorously designed RCTs are required to substantiate these findings.

## Funding

This study was supported by the National Key Research and Development Program‐Elderly Clinical Laboratory Smart Cloud Service Platform and Clinical Laboratory Demonstration (Ly2025jy024); self‐selected topic by the Chinese Academy of Chinese Medical Sciences (zz180524); and The 2025 Special Action on Promoting Traditional Chinese Medicine Health for the “Healthy Yunnan” Initiative of the Yunnan Provincial Health Commission.

## Conflicts of Interest

The authors declare no conflicts of interest.

## Supporting Information

Additional supporting information can be found online in the Supporting Information section.

## Supporting information


**Supporting Information 1** Supporting Information 1 presents the complete search strategy, detailing the search terms and Boolean operators used to identify eligible randomized controlled trials. This information supports the reproducibility of the literature search process and ensures transparency in the study’s methodological approach. Supporting Information 2 contains league Tables for the three primary outcomes—pain relief rate (PRE), Numerical Rating Scale (NRS) scores, and adverse reactions. These tables display the results of all pairwise comparisons between interventions, including effect sizes and 95% confidence intervals, thereby complementing the network meta‐analysis results reported in the main text. Supporting Information 3 includes the results of sensitivity analyses, performed using the leave‐one‐out method for each outcome. These analyses confirm the robustness of the findings by demonstrating that no single study disproportionately influenced the overall effect estimates. Supporting Information 4 summarizes the CINeMA assessments, which evaluate the quality of evidence for each comparison across outcomes. The tables detail the reasons for downgrading evidence quality, such as risk of bias, imprecision, or heterogeneity, offering a transparent appraisal of the confidence in the findings.

**Supporting Information 2** PRISMA_2020_checklist.

## Data Availability

The data that support the findings of this study are available from the corresponding author upon reasonable request.
