# Supplementary material for: Different Acupuncture Combined With Three‐Step Analgesic Therapy in the Treatment of Bone Metastatic Cancer Pain: A Network Meta‐Analysis Based on Randomized Controlled Trials
Source: Pain Res Manag. 2026 Sep 2;2026:7404872. doi: 10.1155/prm/7404872 (PMC13537303; doi:10.1155/prm/7404872)
Supplement: Supplementary file 1 [file PRM-2026-7404872-s001.docx]

**1.Retrieval strategy**

PubMed,Embase,Cochrane Library,CNKI,Wanfang,VIP,SinoMed

| **Step** | **Search term** |
| --- | --- |
| #1 | Acupuncture OR Pharmacopuncture OR Acupotomy OR Acupotomies OR Acupoint* OR Needle OR Acusector OR electrical stimulation OR TAES OR Moxibustion OR Moxabustion［Title/Abstract］ |
| #2 | Cancer Pain OR Cancer Pain* OR Cancer Related Pain OR Cancer Associated Pain OR Neoplasm Related Pain OR Neoplasm Associated Pain OR Oncological Pain* OR Tumor Related Pain OR Tumor Associated Pain OR Oncology Pain*［Title/Abstract］ |
| #3 | Bone* OR Bony Apophyses OR Bony Apophysis OR Condyle*［Title/Abstract］ |
| #4 | random［Title/Abstract］ |
| #5 | #1 AND #2 AND #3 AND #4 |

Web of science

| **Step** | **Search term** |
| --- | --- |
| #1 | Acupuncture OR Pharmacopuncture OR Acupotomy OR Acupotomies OR Acupoint* OR Needle OR Acusector OR electrical stimulation OR TAES OR Moxibustion OR Moxabustion［Topic］ |
| #2 | Cancer Pain OR Cancer Pain* OR Cancer Related Pain OR Cancer Associated Pain OR Neoplasm Related Pain OR Neoplasm Associated Pain OR Oncological Pain* OR Tumor Related Pain OR Tumor Associated Pain OR Oncology Pain*［Topic］ |
| #3 | Bone* OR Bony Apophyses OR Bony Apophysis OR Condyle*［Topic］ |
| #4 | random［Topic］ |
| #5 | #1 AND #2 AND #3 AND #4 |

**2.League table**

(1)Pain relief rate

| **8** | **1** | **2and1** | **5and7and1** | **5and3and1** | **10and1** | **5and1** | **3and1** | **9and1** | **4and3and1** |
| --- | --- | --- | --- | --- | --- | --- | --- | --- | --- |
| 8 | 0.65 (0.46, 0.93) | . | . | . | . | . | . | . | . |
| 0.65 (0.46, 0.93) | 1 | 0.93 (0.72, 1.21) | 0.86 (0.63, 1.20) | 0.84 (0.60, 1.19) | 0.80 (0.55, 1.17) | 0.81 (0.70, 0.93) | 0.75 (0.56, 1.00) | 0.60 (0.37, 0.98) | 0.57 (0.40, 0.81) |
| 0.61 (0.39, 0.94) | 0.93 (0.72, 1.21) | 2and1 | . | . | . | . | . | . | . |
| 0.56 (0.35, 0.91) | 0.86 (0.63, 1.20) | 0.93 (0.61, 1.41) | 5and7and1 | . | . | . | . | . | . |
| 0.55 (0.34, 0.90) | 0.84 (0.60, 1.19) | 0.90 (0.59, 1.39) | 0.97 (0.61, 1.56) | 5and3and1 | . | . | . | . | . |
| 0.52 (0.31, 0.87) | 0.80 (0.55, 1.17) | 0.86 (0.54, 1.36) | 0.92 (0.56, 1.52) | 0.95 (0.57, 1.58) | 10and1 | . | . | . | . |
| 0.52 (0.36, 0.77) | 0.81 (0.70, 0.93) | 0.87 (0.64, 1.17) | 0.93 (0.65, 1.33) | 0.96 (0.66, 1.39) | 1.01 (0.67, 1.51) | 5and1 | . | . | . |
| 0.49 (0.31, 0.77) | 0.75 (0.56, 1.00) | 0.80 (0.54, 1.19) | 0.86 (0.56, 1.33) | 0.89 (0.57, 1.39) | 0.93 (0.58, 1.51) | 0.93 (0.67, 1.28) | 3and1 | . | . |
| 0.39 (0.21, 0.72) | 0.60 (0.37, 0.98) | 0.64 (0.37, 1.13) | 0.69 (0.38, 1.25) | 0.71 (0.39, 1.30) | 0.75 (0.40, 1.40) | 0.74 (0.45, 1.24) | 0.80 (0.45, 1.42) | 9and1 | . |
| 0.37 (0.22, 0.61) | 0.57 (0.40, 0.81) | 0.61 (0.39, 0.95) | 0.66 (0.40, 1.07) | 0.67 (0.41, 1.11) | 0.71 (0.42, 1.20) | 0.70 (0.48, 1.04) | 0.76 (0.48, 1.21) | 0.95 (0.51, 1.74) | 4and3and1 |

(2) NRS

| **3and1** | **6and1** | **5and7and1** | **5and1** | **10and1** | **5and3and1** | **2and1** | **4and3and1** | **1** |
| --- | --- | --- | --- | --- | --- | --- | --- | --- |
| 3and1 | . | . | . | . | . | . | . | -2.30 (-3.37, -1.23) |
| -0.34 (-2.02, 1.34) | 6and1 | . | . | . | . | . | . | -1.96 (-3.26, -0.66) |
| -0.79 (-2.26, 0.68) | -0.45 (-2.09, 1.19) | 5and7and1 | . | . | . | . | . | -1.51 (-2.52, -0.50) |
| -1.04 (-2.22, 0.13) | -0.70 (-2.09, 0.68) | -0.25 (-1.37, 0.86) | 5and1 | . | . | . | . | -1.26 (-1.73, -0.78) |
| -1.40 (-2.92, 0.12) | -1.06 (-2.74, 0.62) | -0.61 (-2.08, 0.86) | -0.36 (-1.53, 0.82) | 10and1 | . | . | . | -0.90 (-1.97, 0.17) |
| -1.55 (-3.04, -0.06) | -1.21 (-2.87, 0.45) | -0.76 (-2.21, 0.69) | -0.51 (-1.65, 0.64) | -0.15 (-1.65, 1.35) | 5and3and1 | . | . | -0.75 (-1.79, 0.29) |
| -2.02 (-3.63, -0.41) | -1.68 (-3.44, 0.08) | -1.23 (-2.79, 0.33) | -0.98 (-2.26, 0.31) | -0.62 (-2.23, 0.99) | -0.47 (-2.06, 1.12) | 2and1 | . | -0.28 (-1.48, 0.92) |
| -2.05 (-3.53, -0.58) | -1.72 (-3.36, -0.07) | -1.27 (-2.70, 0.17) | -1.01 (-2.13, 0.11) | -0.66 (-2.13, 0.82) | -0.50 (-1.96, 0.95) | -0.03 (-1.60, 1.53) | 4and3and1 | -0.24 (-1.26, 0.77) |
| -2.30 (-3.37, -1.23) | -1.96 (-3.26, -0.66) | -1.51 (-2.52, -0.50) | -1.26 (-1.73, -0.78) | -0.90 (-1.97, 0.17) | -0.75 (-1.79, 0.29) | -0.28 (-1.48, 0.92) | -0.24 (-1.26, 0.77) | 1 |

(3)adverse reaction

| **8** | **4and3and1** | **2and1** | **3and1** | **5and3and1** | **5and1** | **10and1** | **1** | **5and7and1** |
| --- | --- | --- | --- | --- | --- | --- | --- | --- |
| 8 | . | . | . | . | . | . | 0.07 (0.03, 0.18) | . |
| 0.37 (0.10, 1.44) | 4and3and1 | . | . | . | . | . | 0.18 (0.07, 0.46) | . |
| 0.23 (0.06, 0.91) | 0.63 (0.17, 2.37) | 2and1 | . | . | . | . | 0.29 (0.11, 0.74) | . |
| 0.16 (0.05, 0.55) | 0.44 (0.14, 1.44) | 0.70 (0.22, 2.29) | 3and1 | . | . | . | 0.41 (0.20, 0.85) | . |
| 0.16 (0.03, 0.79) | 0.42 (0.09, 2.07) | 0.67 (0.14, 3.29) | 0.96 (0.22, 4.17) | 5and3and1 | . | . | 0.43 (0.12, 1.54) | . |
| 0.14 (0.05, 0.41) | 0.38 (0.14, 1.06) | 0.60 (0.22, 1.68) | 0.86 (0.37, 1.98) | 0.90 (0.23, 3.44) | 5and1 | . | 0.48 (0.32, 0.73) | . |
| 0.12 (0.04, 0.34) | 0.33 (0.12, 0.88) | 0.52 (0.19, 1.40) | 0.74 (0.34, 1.63) | 0.77 (0.21, 2.89) | 0.86 (0.51, 1.46) | 10and1 | 0.56 (0.40, 0.77) | . |
| 0.07 (0.03, 0.18) | 0.18 (0.07, 0.46) | 0.29 (0.11, 0.74) | 0.41 (0.20, 0.85) | 0.43 (0.12, 1.54) | 0.48 (0.32, 0.73) | 0.56 (0.40, 0.77) | 1 | 0.67 (0.20, 2.27) |
| 0.05 (0.01, 0.22) | 0.12 (0.03, 0.57) | 0.19 (0.04, 0.90) | 0.27 (0.07, 1.14) | 0.29 (0.05, 1.68) | 0.32 (0.09, 1.17) | 0.37 (0.10, 1.32) | 0.67 (0.20, 2.27) | 5and7and1 |

**3.Sensitivity analysis**

(1)Efficacy

(2)NRS

(3)adverse reaction

**4.CINeMA results**

(1)Pain relief rate

| Comparison | Number of studies | Within-study bias | Reporting bias | Indirectness | Imprecision | Heterogeneity | Incoherence | Confidence rating | Reason(s) for downgrading |
| --- | --- | --- | --- | --- | --- | --- | --- | --- | --- |
| 1:08 | 1 | No concerns | Some concerns | No concerns | No concerns | Major concerns | Some concerns | Very low | ["Reporting bias","Heterogeneity","Incoherence"] |
| 1:10and1 | 1 | Some concerns | Some concerns | No concerns | Major concerns | No concerns | Some concerns | Very low | ["Within-study bias","Reporting bias","Imprecision","Incoherence"] |
| 1:2and1 | 2 | No concerns | Some concerns | No concerns | Major concerns | No concerns | Some concerns | Very low | ["Reporting bias","Imprecision","Incoherence"] |
| 1:3and1 | 2 | No concerns | Some concerns | No concerns | No concerns | Major concerns | Some concerns | Very low | ["Reporting bias","Heterogeneity","Incoherence"] |
| 1:4and3and1 | 2 | No concerns | Some concerns | No concerns | No concerns | No concerns | Some concerns | Low | ["Reporting bias","Incoherence"] |
| 1:5and1 | 7 | No concerns | Some concerns | No concerns | No concerns | Major concerns | Some concerns | Very low | ["Reporting bias","Heterogeneity","Incoherence"] |
| 1:5and3and1 | 1 | No concerns | Some concerns | No concerns | Major concerns | No concerns | Some concerns | Very low | ["Reporting bias","Imprecision","Incoherence"] |
| 1:5and7and1 | 1 | No concerns | Some concerns | No concerns | Major concerns | No concerns | Some concerns | Very low | ["Reporting bias","Imprecision","Incoherence"] |
| 1:9and1 | 1 | No concerns | Some concerns | No concerns | No concerns | Major concerns | Some concerns | Very low | ["Reporting bias","Heterogeneity","Incoherence"] |

(2)NRS

| Comparison | Number of studies | Within-study bias | Reporting bias | Indirectness | Imprecision | Heterogeneity | Incoherence | Confidence rating | Reason(s) for downgrading |
| --- | --- | --- | --- | --- | --- | --- | --- | --- | --- |
| 1:10and1 | 1 | Some concerns | Some concerns | No concerns | No concerns | No concerns | Some concerns | Low | ["Within-study bias","Reporting bias","Incoherence"] |
| 1:2and1 | 1 | No concerns | Some concerns | No concerns | No concerns | Major concerns | Some concerns | Very low | ["Reporting bias","Heterogeneity","Incoherence"] |
| 1:3and1 | 1 | No concerns | Some concerns | No concerns | No concerns | No concerns | Some concerns | Low | ["Reporting bias","Incoherence"] |
| 1:4and3and1 | 1 | No concerns | Some concerns | No concerns | No concerns | No concerns | Some concerns | Low | ["Reporting bias","Incoherence"] |
| 1:5and1 | 6 | No concerns | Some concerns | No concerns | No concerns | No concerns | Some concerns | Low | ["Reporting bias","Incoherence"] |
| 1:5and3and1 | 1 | No concerns | Some concerns | No concerns | No concerns | No concerns | Some concerns | Low | ["Reporting bias","Incoherence"] |
| 1:5and7and1 | 1 | No concerns | Some concerns | No concerns | No concerns | No concerns | Some concerns | Low | ["Reporting bias","Incoherence"] |
| 1:6and1 | 1 | No concerns | Some concerns | No concerns | Major concerns | No concerns | Some concerns | Very low | ["Reporting bias","Imprecision","Incoherence"] |

(3)adverse reaction

| Comparison | Number of studies | Within-study bias | Reporting bias | Indirectness | Imprecision | Heterogeneity | Incoherence | Confidence rating | Reason(s) for downgrading |
| --- | --- | --- | --- | --- | --- | --- | --- | --- | --- |
| 1:08 | 1 | No concerns | Some concerns | No concerns | No concerns | No concerns | Some concerns | Low | ["Reporting bias","Incoherence"] |
| 1:10and1 | 1 | Some concerns | Some concerns | No concerns | No concerns | No concerns | Some concerns | Low | ["Within-study bias","Reporting bias","Incoherence"] |
| 1:2and1 | 2 | No concerns | Some concerns | Some concerns | No concerns | No concerns | Some concerns | Low | ["Reporting bias","Indirectness","Incoherence"] |
| 1:3and1 | 1 | No concerns | Some concerns | No concerns | No concerns | Major concerns | Some concerns | Very low | ["Reporting bias","Heterogeneity","Incoherence"] |
| 1:4and3and1 | 1 | No concerns | Some concerns | No concerns | No concerns | No concerns | Some concerns | Low | ["Reporting bias","Incoherence"] |
| 1:5and1 | 6 | No concerns | Some concerns | No concerns | No concerns | No concerns | Some concerns | Low | ["Reporting bias","Incoherence"] |
| 1:5and3and1 | 1 | No concerns | Some concerns | No concerns | Major concerns | No concerns | Some concerns | Very low | ["Reporting bias","Imprecision","Incoherence"] |
| 1:5and7and1 | 1 | No concerns | Some concerns | No concerns | Major concerns | No concerns | Some concerns | Very low | ["Reporting bias","Imprecision","Incoherence"] |
